# Measuring changes in substrate utilization in the myocardium in response to fasting using hyperpolarized [1-^13^C]butyrate and [1-^13^C]pyruvate

**DOI:** 10.1038/srep25573

**Published:** 2016-05-06

**Authors:** Jessica A. M. Bastiaansen, Matthew E. Merritt, Arnaud Comment

**Affiliations:** 1 Department of Radiology, University Hospital Lausanne (CHUV) and University of Lausanne (UNIL), Lausanne, Switzerland; 2 Laboratory of Functional and Metabolic Imaging, Ecole Polytechnique Fédérale de Lausanne, Lausanne, Switzerland; 3 Department of Biochemistry and Molecular Biology, University of Florida, Gainesville, FL 32610, USA; 4 Institute of Physics of Biological Systems, Ecole Polytechnique Fédérale de Lausanne, Lausanne, Switzerland

## Abstract

Cardiac dysfunction is often associated with a shift in substrate preference for ATP production. Hyperpolarized (HP) ^13^C magnetic resonance spectroscopy (MRS) has the unique ability to detect real-time metabolic changes *in vivo* due to its high sensitivity and specificity. Here a protocol using HP [1-^13^C]pyruvate and [1-^13^C]butyrate is used to measure carbohydrate versus fatty acid metabolism *in vivo*. Metabolic changes in fed and fasted Sprague Dawley rats (n = 36) were studied at 9.4 T after tail vein injections. Pyruvate and butyrate competed for acetyl-CoA production, as evidenced by significant changes in [^13^C]bicarbonate (−48%), [1-^13^C]acetylcarnitine (+113%), and [5-^13^C]glutamate (−63%), following fasting. Butyrate uptake was unaffected by fasting, as indicated by [1-^13^C]butyrylcarnitine. Mitochondrial pseudoketogenesis facilitated the labeling of the ketone bodies [1-^13^C]acetoacetate and [1-^13^C]β-hydroxybutyryate, without evidence of true ketogenesis. HP [1-^13^C]acetoacetate was increased in fasting (250%) but decreased during pyruvate co-injection (−82%). Combining HP ^13^C technology and co-administration of separate imaging agents enables noninvasive and simultaneous monitoring of both fatty acid and carbohydrate oxidation. This protocol illustrates a novel method for assessing metabolic flux through different enzymatic pathways simultaneously and enables mechanistic studies of the changing myocardial energetics often associated with disease.

An emerging treatment avenue for ischemic heart disease is modulation of the substrate preference of the myocardium to improve the production of ATP^1^,^2^,^3^. Substrate selection is a clinically important parameter that varies with the severity of the myocardial pathology and is subject to manipulation by pharmacological intervention^3^,^4^. The long history of measuring substrate preference has yielded many conflicting results, but there is a general consensus that the onset of heart failure is characterized by a decreased utilization of fatty acids, and an increase in carbohydrate metabolism^3^. Measuring substrate selection *in vivo* in a rapid and reproducible manner without ionizing radiation would constitute a fundamental advance in the diagnosis and treatment of heart failure and could help in correctly choosing the most effective intervention in a single patient, i.e., personalized healthcare.

The heart uses a variety of fuel sources to meet its energy requirements, namely fatty acids, ketone bodies, and carbohydrates; the consumption of the latter being up-regulated in the failing heart^5^. Since the complete oxidation of a single glucose molecule provides more molecules of ATP per mole of oxygen than any other substrate^6^, metabolic approaches for increasing cardiac efficiency include suppressing fatty acid oxidation and increasing glucose oxidation^3^. Recently, this hypothesis has been called into question, with the suggestion that insulin resistance in the heart has a cardioprotective effect^7^. With alternative hypotheses for optimizing myocardial function present, new techniques that can measure substrate competition will serve an important purpose in establishing which model of myocardial metabolism is most accurate.

Plasma substrate concentrations can vary dramatically depending on the physiological state of the body and as a result the myocardium modulates its substrate selection in order to maintain high levels of ATP^8^. For instance, fasting leads to an increase of circulating free fatty acids (FFA)^9^, an acceleration of lipid oxidation and a reduction of glycolysis in peripheral tissues^10^, as well as an elevation of ketone body concentrations, which eventually inhibit carbohydrate and fatty acid oxidation^11^,^12^. Acetyl-CoA is the metabolite formed at the crossroads between lipid and carbohydrate metabolism and it is located at the entry of the tricarboxylic acid (TCA) cycle (Fig. 1). It is produced from fatty acids via β -oxidation and from carbohydrates through the glycolytic pathway, via the intramitochondrial pyruvate dehydrogenase (PDH) complex. Substrate selection is regulated by many mechanisms, including the intramitochondrial acetyl-CoA to CoA ratio. An increased ratio results in the inhibition of PDH activity whereas a decreased ratio activates PDH^13^. When acetyl groups are abundant they can be stored as acetylcarnitine, a reaction catalyzed by carnitine acetyl transferase (CAT)^8^.

Recent developments in hyperpolarized ^13^C magnetic resonance (MR) allow the study of real-time metabolism of ^13^C labeled pyruvate, providing a direct measurement of the flux through PDH by the formation of CO_2_ and bicarbonate^14^,^15^,^16^. Hyperpolarized ^13^C pyruvate alone, has been used in promising pre-clinical investigations of myocardial metabolism of the failing heart^17^,^18^,^19^,^20^. The effect of increased fatty acid availability on the metabolism of hyperpolarized pyruvate has been studied in perfused rat heart^14^,^21^, while hyperpolarized [1-^13^C]butyrate was recently proposed as a substrate to study cardiac fatty acid metabolism^22^,^23^. Metabolism of hyperpolarized [1-^13^C]butyrate provides an abundance of metabolic information about its exchange with butyrylCoA via the formation of [1-^13^C]butyrylcarnitine, TCA cycle turnover via the formation of [5-^13^C]citrate, the ^13^C transfer rate between α -ketoglutarate and glutamate through the evolution of the [5-^13^C]glutamate signal, the enzymatic activity of CAT via the formation of [1-^13^C]acetylcarnitine, and a process referred to as pseudoketogenenesis^24^,^25^, via the formation of [1-^13^C]β -hydroxybutyrate (BHB) and [1-^13^C]acetoacetate (AcAc). Note that TCA cycle flux can be probed through the measurement of the [5-^13^C]citrate signal evolution following the injection of hyperpolarized [1-^13^C]acetate^26^, but neither [5-^13^C]citrate nor [5-^13^C]glutamate would be detectable if hyperpolarized [1-^13^C]acetate and [1-^13^C]pyruvate were co-injected because of the overlapping resonances of [1-^13^C]pyruvate hydrate and [1-^13^C]acetate, respectively.

The considerable advantage of the hyperpolarized ^13^C MR method is that substrate selection is monitored by the appearance of specific metabolites produced by pathways leading to acetyl-CoA production and its subsequent entry into the TCA cycle proper. The aim of this study was to measure the change in substrate utilization, *in vivo* and in real-time, in response to an induced shift in metabolism using the co-administration of hyperpolarized [1-^13^C]pyruvate and [1-^13^C]butyrate.

## Results

### Carbohydrate and Fatty Acid Metabolism in the Fed State

Hyperpolarized [1-^13^C]pyruvate metabolism led to the detection of [1-^13^C]lactate, [1-^13^C]alanine, ^13^CO_2_, ^13^C bicarbonate and [1-^13^C]pyruvate hydrate (Fig. 2a). The measured metabolite ratios between total lactate, bicarbonate and alanine relative to pyruvate were 0.049 ±  0.010, 0.027 ±  0.012 and 0.027 ±  0.009 respectively (Fig. 3a–c). The detection of both bicarbonate and CO_2_ in the fed animals allowed us to determine the myocardial pH. A stable value of 7.3 ±  0.1 was observed and demonstrated that the injections did not significantly disturb pH during the experiment (Supplemental Fig. S1).

Butyrate metabolism was similarly informative. The C1 of butyrate has a relatively long longitudinal relaxation time (T_1_) compared to longer chain fatty acids and its resonance signal does not overlap with that of [5-^13^C]glutamate like [1-^13^C]acetate does (Fig. 2b). The following butyrate-derived metabolites were detected *in vivo:* [5-^13^C]glutamate, [1-^13^C]β -hydroxybutyrate, [5-^13^C]citrate, [1-^13^C]acetoacetate, [1-^13^C]butyrylcarnitine, and [1-^13^C]acetylcarnitine (Table 1). The resonance observed at 176.5 ppm was assigned to that of [1-^13^C]butyrylcarnitine based on high resolution ^13^C MR of a mixture containing both ^13^C labeled acetylcarnitine and butyrylcarnitine (Supplemental Fig. S2). Butyrate β -oxidation produces two units of acetyl-CoA, only one of which is labeled and hyperpolarized. As a fraction of the parent butyrate signal, acetylcarnitine (Fig. 4a) was approximately twice the intensity of the glutamate (Fig. 4b) peaks and four times the intensity of the butyrylcarnitine (Fig. 4c) and acetoacetate peaks (Fig. 4d) and showed nearly identical intensities between the fed and fasted state. The normalized fractions of acetylcarnitine, glutamate, butyrylcarnitine, and acetoacetate relative to butyrate were 0.008 ±  0.001, 0.004 ±  0.001, 0.0012 ±  0.0004, and 0.0012 ±  0.0002 respectively in the fed state (Fig. 4).

Substrate competition was clearly evident upon co-injection of the agents. Hyperpolarized ^13^C MRS clearly show the time evolution of both hyperpolarized substrates (Fig. 2c). Although there was some spectral overlap, most metabolites could be detected in the co-injection experiments (Fig. 2d). The competition presented by butyrate results in a decrease in the bicarbonate signal, and a significant increase in the lactate to pyruvate ratio. From the perspective of butyrate metabolism, the co-injection of pyruvate produced multiple signs of competition in the fed state. The acetylcarnitine to butyrate ratio (Fig. 4b), increased upon co-injection, but did not reach statistical significance. The [5-^13^C]glutamate to butyrate ratio (Fig. 4b) decreased significantly upon introduction of pyruvate (0.41 ±  0.06 to 0.08 ±  0.01). Additionally, the co-injection of pyruvate nearly quenched the appearance of acetoacetate derived from butyrate,(0.0012 ±  0.0002 to 0.0002 ±  0.0002) (Fig. 4d).

The fractional contribution of the downstream metabolites to the total detected signal was not significantly different when fasting or in the case of co-injection (Supplemental Fig. S3).

### Carbohydrate and Fatty Acid Metabolism in the Fasted State

Fasting led to significant changes in both the pyruvate and butyrate control experiments and in the substrate selection protocol. Alanine as a fraction of pyruvate signal was largely constant across all injection conditions (Fig. 3c). After an overnight fast, the ratio of bicarbonate to pyruvate reduced significantly in both the single and co-injection groups, and a significant increase in the lactate to pyruvate ratios could be observed (Fig. 3a).

In the butyrate control, a significant increase of the ketone body acetoacetate was observed in both the single and co-injection experiment after fasting (Fig. 4d). The ratio of butyrylcarnitine to butyrate was stable in the fed relative to the fasted cases (Fig. 4c).

Co-injection of pyruvate and butyrate in the fasted state resulted in changes to the downstream metabolites of both metabolic probes compared to the fed animals. The lactate signal rose significantly between the fed and fasted states during co-injection, while the alanine and bicarbonate signals did not show significant changes. From the perspective of butyrate metabolism in the fasted state, the co-injection of pyruvate decreased the glutamate to butyrate ratio (Fig. 4b) and also that of acetoacetate (Fig. 4d). Fasting did not induce a change in the acetylcarnitine to butyrate ratio when comparing the butyrate protocol to the competition protocol (Fig. 4a). Additionally, fasting results in an apparent restoration of the acetoacetate signal even in the presence of pyruvate compared with fed animals (Fig. 4d).

## Discussion

During the switch to a fasting profile, ketone concentrations climb in the plasma, as does the availability of fatty acids. Therefore, in addition to a change in enzyme expression in the myocardium, there is a different profile of circulating substrates available to the heart. The circulating glucose concentration in the fed animals was measured as 9.0 ±  0.7 mM and in the fasted animals it was 4.5 ±  1.0 mM. These changes in substrate availability will affect the signals derived from the hyperpolarized experiments.

**Figure 1:**
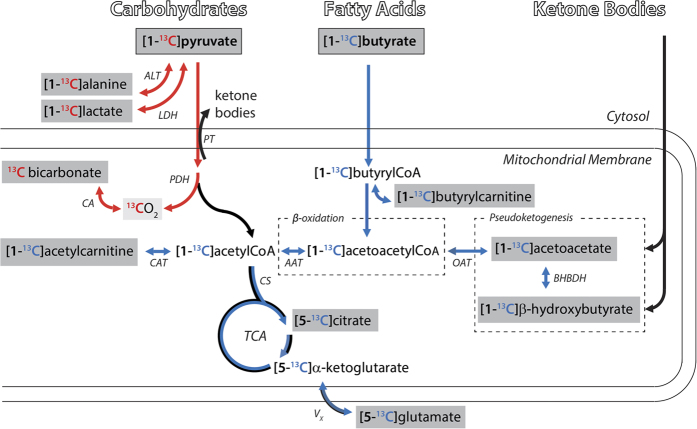
Metabolism of [1-^13^C]pyruvate and [1-^13^C]butyrate in the myocardium *in vivo*. Metabolic scheme indicating the propagation of the ^13^C label from pyruvate to its downstream metabolites with red arrows and those of butyrate with blue arrows. The ^13^C label of [1-^13^C]pyruvate will not enter the TCA cycle. After an overnight fast, ketone body uptake will increase the intracellular acetoacetate and β -hydroxybutyrate concentrations. Detectable metabolite ^13^C resonances are indicated with grey boxes. CAT: carnitine acetyltransferase; CS: citrate synthase; AAT: acetoacetyl-CoA thiolase; OAT: 3-oxoacid CoA transferase; PT: pyruvate transporter; PDH: pyruvate dehydrogenase, LDH: lactate dehydrogenase; ALT: alanine transaminase; BHBDH: β -hydroxybutyrate dehydrogenase; CA: carbonic anhydrase; V_X_: transport and conversion to glutamate.

**Figure 2:**
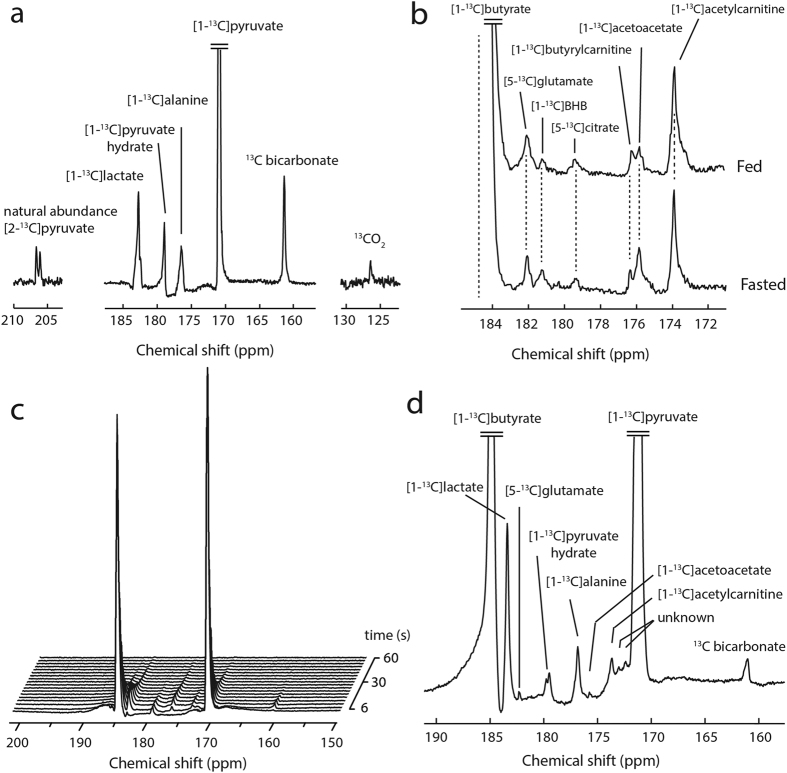
*In vivo* myocardial ^13^C spectra acquired during hyperpolarized MR experiments. (**a**) *In vivo* cardiac ^13^C spectrum recorded after the injection and metabolism of hyperpolarized [1-^13^C]pyruvate in a fed animal. (**b**) *In vivo* cardiac ^13^C spectra recorded after the injection and metabolism of hyperpolarized [1-^13^C]butyrate in the fed and fasted state, without the presence of pyruvate. (**c**) Spectral time course of myocardial metabolism *in vivo* following the co-injection of hyperpolarized [1-^13^C]butyrate and [1-^13^C]pyruvate. (**d**) A sum spectrum with expansion to show the downstream metabolites of HP butyrate and pyruvate in the case of co-injection.

**Figure 3:**
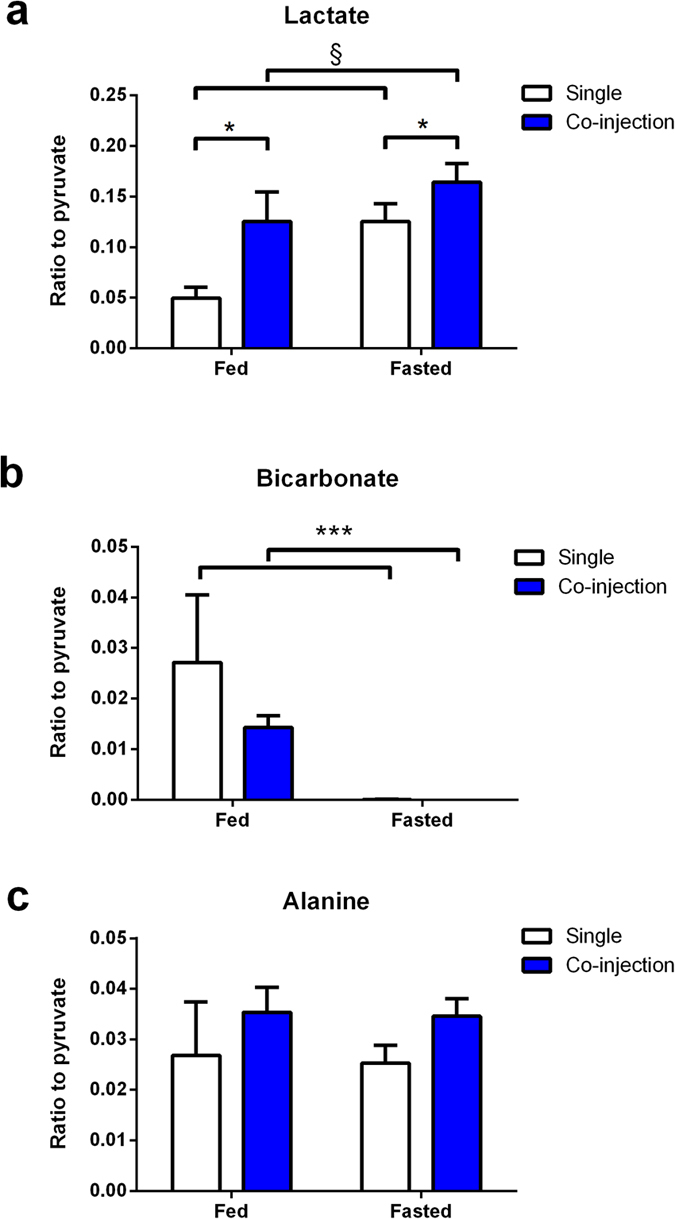
Ratios of measured metabolite signals following hyperpolarized pyruvate metabolism. Signal ratios of lactate (**a**) bicarbonate (**b**) and alanine (**c**) relative to pyruvate where hyperpolarized [1-^13^C]pyruvate was either injected separately or co-injected with hyperpolarized [1-^13^C]butyrate, in both fed and fasted animals. *P =  0.02 in co-injection compared with single injection groups, ^§^P =  0.03 in fasted groups compared with fed groups, ^***^P =  0.001 in fasted groups vs. fed groups.

**Figure 4:**
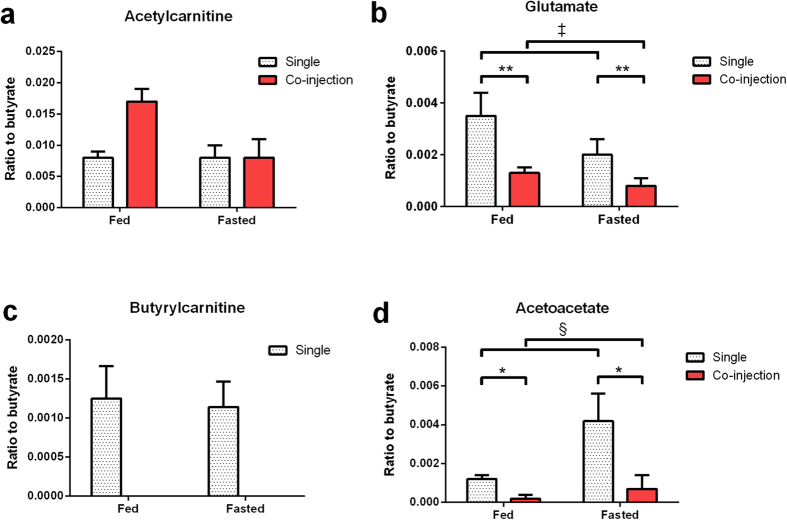
Ratios of measured metabolite signals following hyperpolarized butyrate metabolism. *In vivo* metabolite ratios of ^13^C labeled acetylcarnitine (**a**), glutamate (**b**), butyrylcarnitine (**c**), and acetoacetate (**d**) relative to their hyperpolarized metabolic precursor butyrate. Animals were exposed to a single injection of butyrate, or a co-injection of butyrate and pyruvate. *P =  0.01 in co-injection compared with single injection groups, **P =  0.006 in co-injection compared with single injection groups, ^§^P =  0.05 in fasted groups compared with fed groups, ^‡^P =  0.09 in fasted groups vs. fed groups. Butyrylcarnitine cannot be directly observed during co-injection due to spectral overlap.

**Figure 5:**
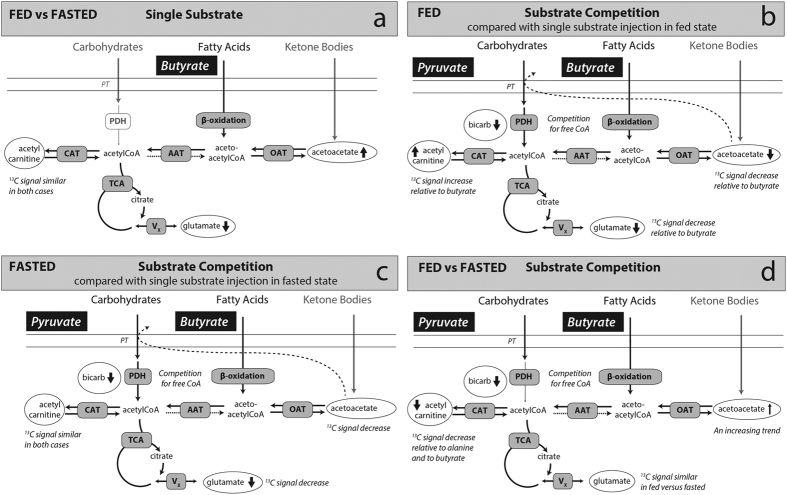
Diagrams of the metabolic pathways probed by the HP experiments. (**a**) Diagram of butyrate metabolism in the fed and fasted case. Fasting lowers the apparent glutamate signal while raising the acetoacetate signal. As carbohydrates and ketones are not labeled, they are grayed out. (**b**) The impact of substrate competition in the fed state. The extra source of acetyl units drives the acetylcarnitine pool size up, while the simultaneous presence of butyrate and pyruvate drives down the appearance of [5-^13^C]glutamate and [^13^C]bicarbonate. It is hypothesized that the decreased acetoacetate signal is ascribed to the effects of antiporting associated with the mitochondrial pyruvate transporter. (**c**) Substrate competition in the fasted state mirrors that of the fed state, though fasting restores some of the [1-^13^C]acetoacetate production. Acetylcarnitine is not significantly different between panels **a** and **c**. (**d**) A comparison of the fed versus fasted state (Panel **b** versus Panel **c**). Fasting produces an increase in the acetoacetate signal but a decrease in the acetylcarnitine signal. Fasting also results in a lower [^13^C]bicarbonate signal whenever it is compared to the fed state.

Pyruvate metabolism largely recapitulated previous studies and is summarized in supplemental information. Like pyruvate, butyrate metabolism is exquisitely sensitive to the nutritional state of the animal. The ^13^C labeling of butyrylcarnitine was stable in both the fed and fasted cases (Fig. 4c), indicating that butyrate transport, reflected by ^13^C labeling of butyrylcarnitine, in the myocardium was not affected by fasting. The acetoacetate resonances were more intense in fasted animals, a direct result of increased ketone body concentrations^10^ and the pseudoketogenic effect^27^. The ^13^C labeling observed in β -hydroxybutyrate and acetoacetate is a result of the exchange between acetoacetyl-CoA and free, unlabeled acetoacetate in the mitochondria, as opposed to true synthesis from two acetyl-CoA precursor units. These ketone bodies were also detected in a perfused heart model injected with hyperpolarized [1-^13^C]butyrate^22^, leading us to believe that they are not an artifact of circulation from the liver. True ketogenesis would result in the ^13^C labeling of [3-^13^C]acetoacetate (Fig. 1), a resonance (~209 ppm) which was never observed. In previous studies using hyperpolarized acetate, which is not β -oxidized, ketone body labeling in the muscle was entirely absent^26^,^28^,^29^,^30^,^31^,^32^, supporting our hypothesis of negligible ^13^C label flux from acetyl-CoA to the ketone bodies in the heart. Acetoacetyl-CoA formation is therefore necessary for observation of β -hydroxybutyrate (Fig. 5a).

Interpretation of the glutamate signal intensities in carbon-13 NMR is subject to a long history of research on the glutamate-oxaloacetate transaminase (GOT) and its interplay with the malate-aspartate shuttle (MAS). Glutamate is ^13^C labeled by exchange with α -ketoglutarate, a pathway alternatively referred to as V_X_^33^ or F1^34^. The glutamate to acetylcarnitine ratio was lower in the fasted compared to fed state while the acetylcarnitine to butyrate ratio remained unchanged, indicating a decrease in glutamate labeling (Fig. 5a). Based on comparisons relative to the butyrate signal, acetylcarnitine was not significantly changed by fasting (Fig. 4a) and the fluxes maintaining the acetylcarnitine pool acting as storage for excess mitochondrial acetyl-CoA do not seem to be affected on the time scale of the experiment^35^. The glutamate pool size remains unchanged for up to a 48 h fast^8^. Therefore it is likely that the decrease in glutamate signal is not a result of a change in glutamate pool size. Substrate competition from circulating, unlabeled nutrients or a change in the equilibration between α -ketoglutarate and glutamate could both be mechanisms underlying the decreased glutamate signal.

Competition between butyrate and pyruvate in the fed animals, compared with a single injection of butyrate or pyruvate, caused a reduction in the observed [^13^C]bicarbonate signal, an increase in the [1-^13^C]lactate signal, a reduction in the [5-^13^C]glutamate signal, a nearly absent [1-^13^C]acetoacetate signal, and an increase in the [1-^13^C]acetylcarnitine signal. All of these observations fit into a detailed narrative of substrate selection between pyruvate and butyrate in the myocardium (Fig. 5b).

The presence of medium or short chain fatty acids can severely restrict the entry of pyruvate into the TCA cycle as well as increase the lactate pool size in perfused hearts^14^. These results are largely duplicated *in vivo*, with the caveat that the pyruvate and butyrate co-injection did not take place over a period long enough to establish a metabolic steady state. High concentrations of pyruvate can partially overcome PDH inhibition by high fatty acid concentrations^36^. Hence, the production of [^13^C]bicarbonate was only halved as opposed to abolished when butyrate was also present, as observed in perfused rat heart^22^, and the lactate signal changed only marginally. Nevertheless these results indicate an almost instantaneous adaptation of myocardial metabolism to substrate availability.

These changes were also a direct reflection of butyrate uptake in the myocardium. The increase in [1-^13^C]acetylcarnitine and the decrease of the [5-^13^C]glutamate and [1-^13^C]acetoacetate signals indicate competition from pyruvate for the limited number of free CoA units in the mitochondria (Fig. 5a). Acetylcarnitine serves as a buffer for extra acetyl units that exceed the capacity of the TCA cycle for oxidation and this circulation of acetyl groups is a fast, well equilibrated process^37^,^38^,^39^. When pyruvate was co-injected, free CoA was rapidly reacted to acetyl-CoA, and the extra acetyl units derived from butyrate oxidation were stored by reaction with carnitine, resulting in the increased observed acetylcarnitine. The lowering of the [5-^13^C]glutamate signal can be accounted for by the same competitive phenomena; [1-^13^C]pyruvate cannot produce labeling of glutamate in the myocardium. However, identification of the mechanism(s) underlying the decreased glutamate signal upon co-injection will require further studies due to the considerations regarding glutamate-α -ketoglutarate exchange.

The mitochondrial pyruvate transporter preferentially antiports acetoacetate over any ion except hydroxide^40^,^41^. Upon co-injection of pyruvate, we hypothesize that the consequent lack of free, unlabeled acetoacetate available for exchange short-circuits the process of pseudoketogenesis. Further experiments, for example blocking the mitochondrial pyruvate transporter, would be required to confirm this hypothesis, but this explanation seems straightforward given the previous work^40^.

In the case of fasting, co-injection resulted in a similar set of results to the fed control, but with some notable differences. Similar to the results with pyruvate only, fasting quenched the appearance of [^13^C]bicarbonate. Compared to the fed state, the sum of the products of hyperpolarized [1-^13^C]butyrate metabolism trended lower (Supplemental Fig. S3). But, the acetylcarnitine signal was the only driver of this difference. Fasting caused a significant increase in the signal of acetoacetate. Given what is known about the increase in concentration of ketone bodies in fasting, the nominal increase in acetoacetate signal is most likely due to restored free acetoacetate availability and increased pseudoketogenesis (Fig. 5d). Further experiments varying the dose of pyruvate should serve to confirm this explanation. The near equivalence of the glutamate signal between the fed and fasted co-injection depends upon both TCA cycle turnover and V_x_. Interpretation of the data would demand other independent measures of both pathways.

In summary, two separate agents, hyperpolarized [1-^13^C]pyruvate and hyperpolarized [1-^13^C]butyrate, were used as controls for a new protocol that measures substrate selection by the simultaneous injection of both agents. The substrate competition experiment has been shown capable of measuring carbohydrate versus fatty acid utilization in the *in vivo* heart, as evidenced by statistically significant changes in the [^13^C]bicarbonate signal (evidence of fatty acid competition with pyruvate) and in glutamate and acetoacetate signals (evidence of pyruvate displacement of fatty acids for the production of acetyl-CoA). These experiments demonstrate that the chemical selectivity inherent to magnetic resonance, when paired with the sensitivity enhancement of hyperpolarization, can be harnessed to produce a protocol that assesses substrate selection in real time, *in vivo*, in the functioning heart.

No changes were observed between fed and fasted states *in vivo* in a recent study using hyperpolarized [1-^13^C]butyrate^22^ whereas many significant differences were detected in the present study. This can likely be attributed to increased spectral resolution at the higher magnetic field strength of 9.4 T used here. The clinical potential of the use of ^13^C hyperpolarized magnetic resonance techniques to probe *in vivo* metabolism has been recently discussed and the first trials in humans for application in cancer monitoring and diagnosis has already been completed^42^. Applications in cardiology also seem at least as promising, and are expected to affect both preclinical and clinical practice^43^. The protocol suggested here should augment the usefulness of hyperpolarized imaging further.

### Biological significance

Pyruvate has been suggested as a treatment for heart failure, where its administration has been shown to increase contractility. Mechanistically, this increase in function was attributed to increased transport of Ca^2+^ ions^44^. However, as aforementioned, pyruvate produces more equivalents of ATP per mole of O_2_ consumed than any other substrate^6^. Due to the superior sensitivity and time resolution (1 s or even lower) available with hyperpolarized MR, it is possible to observe kinetic processes *in vivo* that were previously amenable to study only in cell culture models. If the mitochondrial pyruvate carrier indeed antiports acetoacetate and other competing substrates^40^,^41^, this would alter myocardial substrate selection for acetyl-CoA production. We propose that the beneficial effects of pyruvate administration are not only mediated by Ca^2+^ efflux but also by optimization of the myocardial efficiency as promoted by the export of substrates that compete with pyruvate for oxidation. This hypothesis is amenable to testing in the perfused heart and suggests concurrent treatment with pyruvate and PDH kinase inhibitors should maximally increase energy availability in the myocardium, perhaps as an acute treatment for myocardial infarction or failure.

### Study limitations

Hyperpolarization is limited by the ^13^C longitudinal relaxation time (T_1_) inherent to the molecules used for the studies^45^. While glucose would be a more obvious choice for this protocol, the additional enzymatic steps associated with glycolysis along with the short ^13^C T_1_ in all carbon positions of the glucose molecule greatly reduce the signal intensity of the metabolites relevant for the study of substrate competition. Our choice of [1-^13^C]pyruvate, which has a T_1_ at 9.4 T of ~45 s, implies that we are not measuring glycolytic metabolism, except indirectly through exchange with lactate. Lactate could also be used as a substitute for pyruvate, but the T_1_ of [1-^13^C]lactate is ~25 s in solution, considerably shorter than pyruvate. A similar rationale led us to choose butyrate as the surrogate for fatty acid oxidation. Butyrate is a short chain fatty acid that does not have to be solubilized with albumin, a known source of T_1_ relaxation^21^. Binding of a compound, including any long chain fatty acid tested, to albumin is sufficient to destroy the hyperpolarized signal (data not shown). Other water soluble fatty acids like [1-^13^C]octanoate could have been used^46^, but the increased number of unlabeled carbons subject to β -oxidation would have further halved the signals associated with fatty acid utilization without providing any information on the activity of fatty acid transporters like carnitine palmitoyl transferase I (CPT-I)^13^. The use of [1-^13^C]acetate would ensure a 100% transfer of labeled carbons, and the detection of the TCA cycle intermediate citrate, but it does not follow β -oxidation and the resonance of [1-^13^C]acetate overlaps with that of [5-^13^C]glutamate^26^,^30^. The lack of facilitated transport of butyrate as butyrylcarnitine by CPT-I is the primary drawback of butyrate as a molecular imaging agent, as CPT-I is a primary point of regulation for fatty acid oxidation^13^. Despite its limitations, the use of the short chain fatty acid butyrate still provides unprecedented data about substrate competition and β -oxidation, as evidenced by the data presented here. Although CPT-I is bypassed and no information can be obtained related to the long chain fatty acid transport process itself, the insights gained are still expected to be diagnostic of myocardial dysfunction.

Some objections have been raised to the high concentrations of the exogenous agents used for these experiments, with claims that bolus administration can produce flux through pathways due to thermodynamic effects. However, the short T_1_ of the hyperpolarized carbons works in *the favor* of the experiment. Before pool sizes can change noticeably from the challenge of the injected substrates, the experiment is over. From a safety perspective, high concentrations of injected pyruvate have been shown safe in the first human hyperpolarized imaging experiments^42^ with injected doses up to 0.10 mmol/kg. We have no reason to believe butyrate would not have a similar safety profile.

## Conclusions

The combination of HP pyruvate and butyrate provides unprecedented insight into the selection of carbohydrates and fatty acids for oxidation in the myocardium in real-time, and enables a noninvasive and simultaneous measurement of different enzymatic pathways. The co-injections of hyperpolarized metabolic fuels was sensitive to a simple metabolic perturbation and is thus promising for mechanistic studies of myocardial energetics. The results shown here lead us to hypothesize that injection of a bolus of exogenous pyruvate might increase cardiac function not only due to Ca^2+^ modulation but also due to the export of substrates less efficient than pyruvate for ATP production.

## Methods

### Animals

All studies were performed between 10 am and 2 pm, and all animal experiments were conducted according to federal ethical guidelines. The study was approved by the EPFL Animal Care and Use Committee. Wild-type male Sprague Dawley rats (n =  36, 281 ±  26 g) were anesthetized with 1.5% isoflurane in oxygen. Catheters were placed into the femoral vein for substrate delivery and in the artery to monitor the blood pressure used for cardiac triggering, as previously described^26^,^30^,^47^. The respiration rate, cardiac rhythm, and temperature were monitored and maintained stable, and were not affected by the injections. Plasma glucose concentrations were quantified with the glucose oxidase method using two multi-assay analyzers (GW7 Micro-Stat, Analox Instruments, London, UK). Animals were exposed to an injection of hyperpolarized butyrate (n =  12), pyruvate (n =  10) or both (n =  14) and each group was divided to be in a fed or fasted state (Table 2). Six different groups were studied and each animal was injected once. The animals were fasted overnight.

### Sample preparation and hyperpolarization protocol

Sodium [1-^13^C]butyrate and sodium [1-^13^C]pyruvate (Sigma-Aldrich, Buchs, Switzerland), were hyperpolarized for 2.5 h in a 5 T custom-designed DNP polarizer^48^,^49^. Substrates were prepared separately using TEMPO nitroxyl radicals as previously described^26^,^29^, and were combined in the polarizer to yield identical concentrations of both ^13^C labeled pyruvate and butyrate. Using an automated process^50^ the sample was rapidly dissolved and transferred within 2 s following dissolution to a separator/infusion pump, containing 0.6 mL of phosphate buffered saline and heparin, located inside the bore of the MR scanner. Subsequently, 800 μ L of a 70 mM ^13^C-labeled hyperpolarized substrate solution was automatically infused into the animal, yielding an average injected substrate dose of 0.19 mmol/kg. Typical polarization levels were 13 ±  2%.

### *In vivo* magnetic resonance imaging and spectroscopy

Measurements were carried out in a horizontal bore 9.4 T magnet (Magnex Scientific, Oxford, UK) with a Direct Drive spectrometer (Agilent, Palo Alto, CA, USA). A custom-made radiofrequency (RF) hybrid probe, consisting of a 10 mm diameter proton surface coil and a pair of 10 mm diameter ^13^C surface coils in quadrature mode, was positioned over the chest of the rat for transmission and reception. A hollow glass sphere with a 3 mm inner diameter (Wilmad-LabGlass, NJ, USA) was filled with an aqueous 1 M [1-^13^C]glucose solution and used to adjust the radiofrequency (RF) excitation pulse power and set the reference frequency. Acquisition of gradient echo proton images confirmed the correct placement of the coil and was used to determine the voxel used for shimming.

Cinematographic images (field of view =  40 ×  40 mm^2^; matrix size: 256 ×  256; repetition time T_R_ =  140 ms; echo time T_E_ =  4.5 ms; number of averages =  8; number of frames: 14; slice thickness: 1 mm) were acquired to confirm and set the timing of the cardiac trigger in the end-diastolic phase. The cardiac trigger was typically sent 50 or 60 ms after the maximum observed blood pressure. Cardiac triggered and respiratory gated shimming was performed using the FAST(EST)MAP gradient shimming routine^51^ to reduce the localized proton line width in a myocardial voxel of 4 ×  5 ×  5 mm^3^ to 20–30 Hz acquired with stimulated echo localized spectroscopy (STEAM), resulting in a non-localized proton line width of 80–120 Hz. The MR console was triggered to start acquisition at the beginning of the automated injection process. Series of single pulse acquisitions were sequentially recorded using 30° adiabatic RF excitation pulses (BIR-4)^52^, with ^1^H decoupling using WALTZ^53^. Free induction decays were acquired with 4129 complex data points over a 20 kHz bandwidth. All acquisitions were cardiac triggered and respiratory gated, resulting in a nominal T_R_ between 3 and 3.5 s. The adiabatic pulse offset and power were calibrated to ensure that the RF excitation angle θ  =  30° for all observed metabolites in the entire tissue of interest, and was sufficient to excite the entire range of observable resonances from 100 to 220 ppm. Following *in vivo* data acquisition, a 200 μ L liquid sample was extracted from the separator/infusion pump to retrospectively determine the precise injected substrate concentrations.

### Data analysis

The ^13^C MR spectra were summed and analyzed with Bayes (Washington University, St. Louis, MO, USA) as previously described and metabolite resonance areas were calculated^26^,^47^. All metabolite areas were normalized to their hyperpolarized injected precursor to determine specific metabolite ratios.

### Statistical Methods

Statistics on the metabolite ratios were computed via two-way unbalanced ANOVA, considering the factors of “nutritional state” and “injected substrates”. In case the ANOVA analysis reported significant interaction between the two factors, specific instances were further analyzed via two-tailed student t-tests for unpaired data with equal variance. All data were expressed as mean ±  standard error of the mean (SEM).

## Additional Information

**How to cite this article**: Bastiaansen, J. A. M. *et al.* Measuring changes in substrate utilization in the myocardium in response to fasting using hyperpolarized [1-^13^C]butyrate and [1-^13^ C]pyruvate. *Sci. Rep.* **6**, 25573; doi: 10.1038/srep25573 (2016).

## Supplementary Material

Supplementary Information

## Figures and Tables

**Table 1:** Chemical shifts of observed metabolites following the injection of hyperpolarized sodium [1-^13^C]butyrate and sodium [1-^13^C]pyruvate.

Metabolite	Chemical shift (ppm)
[1-^13^C]butyrate	185.0
[1-^13^C]lactate	183.5
[5-^13^C]glutamate	182.4
[1-^13^C]β -hydroxybutyrate	181.6
[1-^13^C]pyruvate hydrate	179.8
[5-^13^C]citrate	179.7
[1-^13^C]alanine	177.0
[1-^13^C]butyrylcarnitine	176.5
[1-^13^C]acetoacetate	176.0
[1-^13^C]acetylcarnitine	173.9
[1-^13^C]pyruvate	171.4
^13^C bicarbonate	161.3
^13^CO_2_	126.0

**Table 2:** Number of animals studied under each condition.

Subject Groups (# animals)	[1-^13^C]Butyrate	[1-^13^C]Butyrate^+^ [1-^13^C]Pyruvate	[1-^13^C]Pyruvate
Fed	6	7	5
Fasted	6	7	5

## References

[b1] Lopaschuk G. D., Rebeyka I. M. & Allard M. F. Metabolic modulation: a means to mend a broken heart. Circulation 105, 140–142 (2002). 11790689

[b2] Nagoshi T., Yoshimura M., Rosano M. C., Lopaschuk G. D. & Mochizuki S. Optimization of Cardiac Metabolism in Heart Failure. Curr Pharm Des 17, 3846–3853, doi: 10.2174/138161211798357773 (2011). 21933140 PMC3271354

[b3] Neubauer S. The failing heart–an engine out of fuel. N Engl J Med 356, 1140–1151 (2007). 17360992 10.1056/NEJMra063052

[b4] Kantor P. F., Lucien A., Kozak R. & Lopaschuk G. D. The Antianginal Drug Trimetazidine Shifts Cardiac Energy Metabolism From Fatty Acid Oxidation to Glucose Oxidation by Inhibiting Mitochondrial Long-Chain 3-Ketoacyl Coenzyme A Thiolase. Circ Res 86, 580–588, doi: 10.1161/01.res.86.5.580 (2000). 10720420

[b5] Carvajal K. & Moreno-Sanchez R. Heart metabolic disturbances in cardiovascular diseases. Arch Med Res 34, 89–99 (2003). 12700003 10.1016/S0188-4409(03)00004-3

[b6] Malloy C. R., Jones J. G., Jeffrey F. M., Jessen M. E. & Sherry A. D. Contribution of various substrates to total citric acid cycle flux and ]anaplerosis as determined by ^13^C isotopomer analysis and O_2_ consumption in the heart. MAGMA 4, 35–46, doi: 10.1007/bf01759778 (1996). 8774000

[b7] Taegtmeyer H., Beauloye C., Harmancey R. & Hue L. Insulin resistance protects the heart from fuel overload in dysregulated metabolic states. Am J Physiol Heart Circ Physiol 305, H1693–H1697 (2013). 24097426 10.1152/ajpheart.00854.2012 PMC3882545

[b8] Taegtmeyer H. Six blind men explore an elephant: aspects of fuel metabolism and the control of tricarboxylic acid cycle activity in heart muscle. Basic Res Cardiol 79, 322–336 (1984). 6477383 10.1007/BF01908033

[b9] Barrett A. M. Adventitious Factors Affecting Concentration of Free Fatty Acids in Plasma of Rats. Brit J Pharm Chemoth 22, 577–584 (1964). 10.1111/j.1476-5381.1964.tb01711.x PMC1703944 14211687

[b10] Kraupp O., Adlerkas. L., Niessner H. & Plank B. Effects of Starvation and of Acute and Chronic Alloxan Diabetes on Myocardial Substrate Levels and on Liver Glycogen in Rat *in Vivo*. Eur J Biochem 2, 197–214 (1967). 6078532 10.1111/j.1432-1033.1967.tb00126.x

[b11] Olson R. E. Effect of Pyruvate and Acetoacetate on Metabolism of Fatty Acids by Perfused Rat Heart. Nature 195, 597–599, doi: 10.1038/195597b0 (1962). 14481944

[b12] Bassenge E. *et al.* Effect of Ketone Bodies on Cardiac Metabolism. Am J Physiol 208, 162–168 (1965). 14253143 10.1152/ajplegacy.1965.208.1.162

[b13] Stanley W. C., Recchia F. A. & Lopaschuk G. D. Myocardial substrate metabolism in the normal and failing heart. Physiol Rev 85, 1093–1129, doi: 10.1152/physrev.00006.2004 (2005). 15987803

[b14] Merritt M. E. *et al.* Hyperpolarized ^13^C allows a direct measure of flux through a single enzyme-catalyzed step by NMR. Proc Natl Acad Sci USA 104, 19773–19777, doi: 10.1073/pnas.0706235104 (2007). 18056642 PMC2148374

[b15] Golman K. *et al.* Cardiac metabolism measured noninvasively by hyperpolarized ^13^C MRI. Magn Reson Med 59, 1005–1013 (2008). 18429038 10.1002/mrm.21460

[b16] Schroeder M. A. *et al.* *In vivo* assessment of pyruvate dehydrogenase flux in the heart using hyperpolarized carbon-13 magnetic resonance. Proc Natl Acad Sci USA 105, 12051–12056, doi: 10.1073/pnas.0805953105 (2008). 18689683 PMC2515222

[b17] Yoshihara H., Bastiaansen J. A. M., Berthonneche C., Comment A. & Schwitter J. Assessing ischemic myocardial metabolism *in vivo* with hyperpolarized ^13^C: relating the metabolic perturbation to the area at risk. J Cardiovasc Magn Reson 17, 1–2, doi: 10.1186/1532-429X-17-S1-O97 (2015).25589308

[b18] Schroeder M. A. *et al.* Hyperpolarized ^13^C magnetic resonance reveals early- and late-onset changes to *in vivo* pyruvate metabolism in the failing heart. Eur J Heart Fail 15, 130–140, doi: 10.1093/eurjhf/hfs192 (2013). 23258802 PMC3547367

[b19] Dodd M. S. *et al.* Impaired *In Vivo* Mitochondrial Krebs Cycle Activity Following Myocardial Infarction Assessed Using Hyperpolarized Magnetic Resonance Spectroscopy. Circ Cardiovasc Imaging, doi: 10.1161/CIRCIMAGING.114.001857 (2014). PMC4450075 25201905

[b20] Yoshihara H. A., Bastiaansen J. A., Berthonneche C., Comment A. & Schwitter J. An Intact Small Animal Model of Myocardial Ischemia-Reperfusion: Characterization of Metabolic Changes by Hyperpolarized ^13^C MR Spectroscopy. Am J Physiol Heart Circ Physiol, (2015). 10.1152/ajpheart.00376.2015 26453328

[b21] Moreno K. X., Sabelhaus S. M., Merritt M. E., Sherry A. D. & Malloy C. R. Competition of pyruvate with physiological substrates for oxidation by the heart: implications for studies with hyperpolarized [1-(13)C]pyruvate. Am J Physiol-Heart C 298, H1556–H1563, doi: 10.1152/ajpheart.00656.2009 (2010). PMC2867437 20207817

[b22] Ball D. R. *et al.* Hyperpolarized butyrate: a metabolic probe of short chain fatty acid metabolism in the heart. Magn Reson Med 71, 1663–1669, doi: 10.1002/mrm.24849 (2014). 23798473 PMC4238803

[b23] Bastiaansen J. A., Merritt M. E. & Comment A. Real time measurement of myocardial substrate selection *in vivo* using hyperpolarized 13C magnetic resonance. J Cardiovasc Magn Reson 17, O15, doi: 10.1186/1532-429X-17-S1-O15 (2015).

[b24] Des Rosiers C. *et al.* Interference of 3-hydroxyisobutyrate with measurements of ketone body concentration and isotopic enrichment by gas chromatography-mass spectrometry. Anal Biochem 173, 96–105 (1988). 3189805 10.1016/0003-2697(88)90165-0

[b25] Fink G. *et al.* Pseudoketogenesis in the perfused rat heart. J Biol Chem 263, 18036–18042 (1988). 3056937

[b26] Bastiaansen J. A., Cheng T., Lei H., Gruetter R. & Comment A. Direct noninvasive estimation of myocardial tricarboxylic acid cycle flux *in vivo* using hyperpolarized C magnetic resonance. J Mol Cell Cardiol 87, 129–137, doi: 10.1016/j.yjmcc.2015.08.012 (2015). 26297113

[b27] Fink G. *et al.* Pseudoketogenesis in the Perfused Rat-Heart. J Biol Chem 263, 18036–18042 (1988). 3056937

[b28] Jensen P. R. *et al.* Tissue-specific short chain fatty acid metabolism and slow metabolic recovery after ischemia from hyperpolarized NMR *in vivo*. J Biol Chem 284, 36077–36082, doi: 10.1074/jbc.M109.066407 (2009). 19861411 PMC2794723

[b29] Bastiaansen J. A. M. *et al.* *In vivo* enzymatic activity of acetylCoA synthetase in skeletal muscle revealed by 13C turnover from hyperpolarized [1-^13^C]acetate to [1-^13^C]acetylcarnitine. Biochim Biophys Acta 1830, 4171–4178, doi: 10.1016/j.bbagen.2013.03.023 (2013). 23545238

[b30] Bastiaansen J. A. M., Cheng T., Gruetter R. & Comment A. Proceedings of the 20th annual meeting ISMRM, Melbourne, Australia 20, 4324 (2012).

[b31] Flori A. *et al.* Real-time cardiac metabolism assessed with hyperpolarized [1- C]acetate in a large-animal model. Contrast Media Mol Imaging, doi: 10.1002/cmmi.1618 (2014). PMC4362963 25201079

[b32] Koellisch U. *et al.* Metabolic imaging of hyperpolarized [1- C]acetate and [1- C]acetylcarnitine–investigation of the influence of dobutamine induced stress. Magn Reson Med, doi: 10.1002/mrm.25485 (2014). 25298189

[b33] Malloy C. R., Sherry A. D. & Jeffrey F. M. Analysis of tricarboxylic acid cycle of the heart using ^13^C isotope isomers. Am J Physiol 259, H987–995 (1990). 1975735 10.1152/ajpheart.1990.259.3.H987

[b34] Yu X. *et al.* Kinetic analysis of dynamic ^13^C NMR spectra: metabolic flux, regulation, and compartmentation in hearts. Biophysl J 69, 2090–2102 (1995). 10.1016/S0006-3495(95)80080-9 PMC1236443 8580353

[b35] Bohmer T., Norum K. R. & Bremer J. Relative Amounts of Long-Chain Acylcarnitine Acetylcarnitine and Free Carnitine in Organs of Rats in Different Nutritional States and with Alloxan Diabetes. Biochimica et biophysica acta 125, 244–251 (1966).

[b36] Olson M. S., Dennis S. C., Debuysere M. S. & Padma A. Regulation of Pyruvate-Dehydrogenase in Isolated Perfused Rat-Heart. J Biol Chem 253, 7369–7375 (1978). 701258

[b37] Fritz I. B., Schultz S. K. & Srere P. A. Properties of Partially Purified Carnitine Acetyltransferase. J Biol Chem 238, 2509–2517 (1963). 13963148

[b38] Pearson D. J. & Tubbs P. K. Carnitine and Derivatives in Rat Tissues. Biochem J 105, 953–963 (1967). 16742571 10.1042/bj1050953 PMC1198413

[b39] Schroeder M. A. *et al.* The Cycling of Acetyl-Coenzyme A Through Acetylcarnitine Buffers Cardiac Substrate Supply A Hyperpolarized C-13 Magnetic Resonance Study. Circ Cardiovasc Imaging 5, 201–U282 (2012). 22238215 10.1161/CIRCIMAGING.111.969451 PMC3378498

[b40] Halestrap A. P. Pyruvate and ketone-body transport across the mitochondrial membrane. Exchange properties, pH-dependence and mechanism of the carrier. Biochem J 172, 377–387 (1978). 28726 10.1042/bj1720377 PMC1185711

[b41] Paradies G. Interaction of Alpha-Cyano-Cinnamate-C-14 with the Mitochondrial Pyruvate Translocator. Biochim Biophys Acta 766, 446–450 (1984). 6466654 10.1016/0005-2728(84)90260-3

[b42] Nelson S. J. *et al.* Metabolic Imaging of Patients with Prostate Cancer Using Hyperpolarized [1-^13^C]Pyruvate. Sci Transl Med 5, 198ra108, doi: 10.1126/scitranslmed.3006070 (2013). PMC4201045 23946197

[b43] Malloy C. R., Merritt M. E. & Sherry A. D. Could (13)C MRI assist clinical decision-making for patients with heart disease ? NMR Biomed 24, 973–979, doi: 10.1002/Nbm.1718 (2011). 21608058 PMC3174329

[b44] Hasenfuss G. *et al.* Influence of pyruvate on contractile performance and Ca(2+ ) cycling in isolated failing human myocardium. Circulation 105, 194–199 (2002). 11790700 10.1161/hc0202.102238

[b45] Comment A. & Merritt M. E. Hyperpolarized magnetic resonance as a sensitive detector of metabolic function. Biochem 53, 7333–7357, doi: 10.1021/bi501225t (2014). 25369537 PMC4255644

[b46] Yoshihara H. *et al.* Myocardial fatty acid metabolism probed with hyperpolarized [1-^13^C]octanoate. J Cardiovasc Magn Reson 17, O101 (2015).

[b47] Bastiaansen J. A. M., Yoshihara H. A. I., Takado Y., Gruetter R. & Comment A. Hyperpolarized ^13^C lactate as a substrate for *in vivo* metabolic studies in skeletal muscle. Metabolomics 10, 986–994, doi: 10.1007/s11306-014-0630-5 (2014).

[b48] Comment A. *et al.* Design and performance of a DNP prepolarizer coupled to a rodent MRI scanner. Concepts Magn Reson Part B 31B, 255–269 (2007).

[b49] Jannin S. *et al.* A 140 GHz prepolarizer for dissolution dynamic nuclear polarization. J Chem Phys 128, 241102, doi: 10.1063/1.2951994 (2008). 18601309

[b50] Cheng T. *et al.* Automated transfer and injection of hyperpolarized molecules with polarization measurement prior to *in vivo* NMR. NMR Biomed 26, 1582–1588, doi: 10.1002/nbm.2993 (2013). 23893539

[b51] Gruetter R. & Tkac I. Field mapping without reference scan using asymmetric echo-planar techniques. Magn Reson Med 43, 319–323 (2000). 10680699 10.1002/(sici)1522-2594(200002)43:2<319::aid-mrm22>3.0.co;2-1

[b52] Staewen R. S. *et al.* 3-D Flash Imaging Using a Single Surface Coil and a New Adiabatic Pulse, Bir-4. Invest Radiol 25, 559–567 (1990). 2345088 10.1097/00004424-199005000-00015

[b53] Shaka A. J., Keeler J., Frenkiel T. & Freeman R. An Improved Sequence for Broad-Band Decoupling–Waltz-16. J Magn Reson 52, 335–338 (1983).

