# Supplementary material for: Measuring changes in substrate utilization in the myocardium in response to fasting using hyperpolarized [1-13C]butyrate and [1-13C]pyruvate
Source: Sci Rep. 2016 May 6;6:25573. doi: 10.1038/srep25573 (PMC4858671; doi:10.1038/srep25573)
Supplement: Supplementary Information [file srep25573-s1.doc]

**SUPPLEMENTAL INFORMATION FOR:**

**Measuring changes in substrate utilization in the myocardium in response to fasting using hyperpolarized [1-^13^C]butyrate and [1-^13^C]pyruvate**

Jessica AM Bastiaansen, Matthew E Merritt, Arnaud Comment

**Supplemental Table 1.** Average glucose levels and body weight of all animals used in this study

| **Parameter** | **Fed** | **Fasted** |
| --- | --- | --- |
| Body weight [g] | 288 ± 18 | 265 ± 22 |
| Glucose [mM] | 9.0 ± 0.7 | 4.5 ± 1.0 |

**Supplemental Figure S1.** Integral time course of the ^13^C labeled bicarbonate and carbon dioxide signals. The point by point ratio of these biomolecules were used to calculate the pH by applying the Henderson-Hasselbach equation as shown before .

**
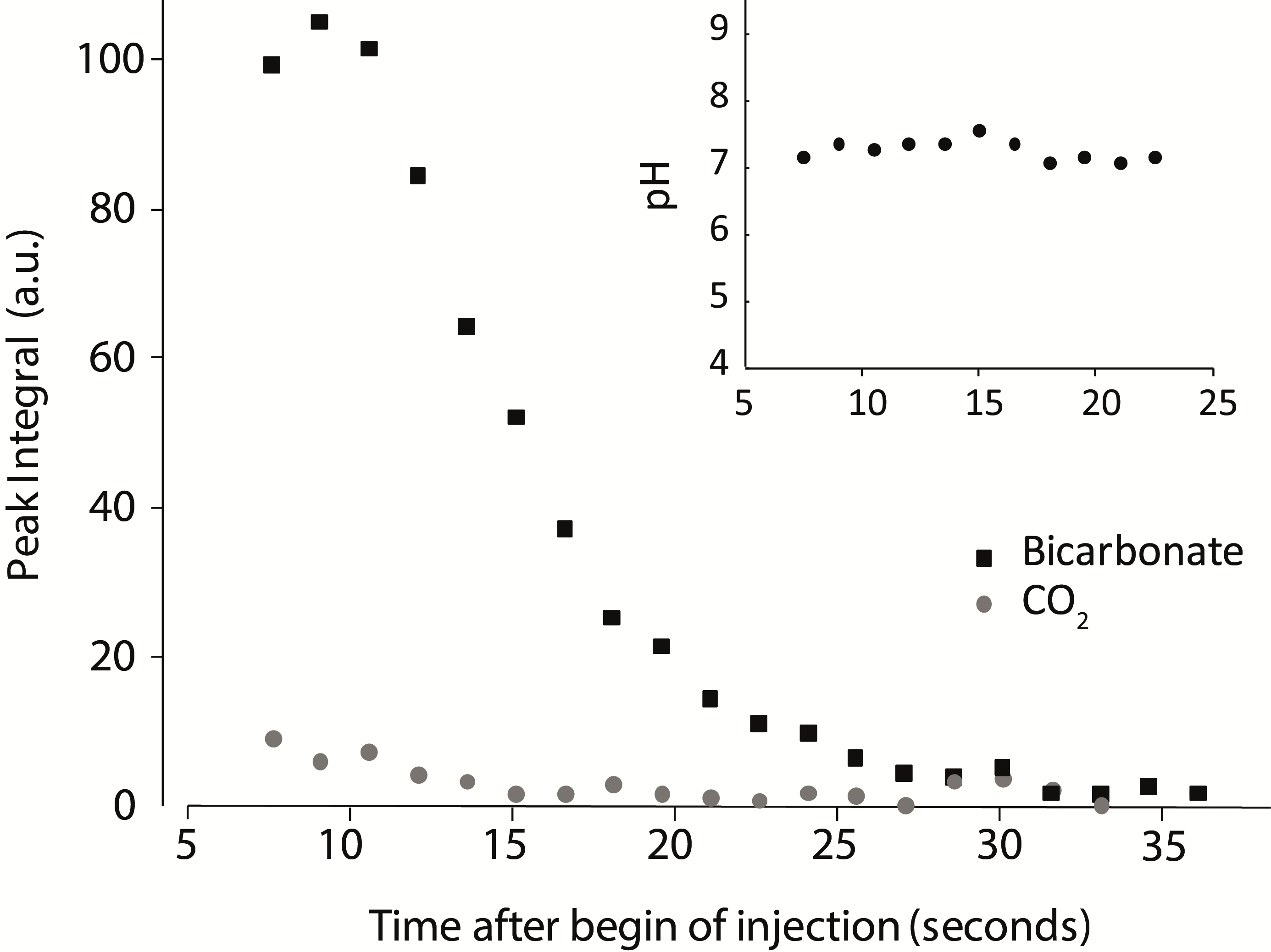
**

**Supplemental Figure S2.** High resolution ^13^C NMR spectrum of butyrylcarnitine and [1-^13^C]acetylcarnitine in D_2_O at pH = 7.0 to assign the butyrylcarnitine resonances observed in the myocardium in vivo. The measurement was performed at 14.1 T using a DRX-600 spectrometer equipped with a 5-mm cryoprobe (bruker BioSpin SA, Fallanden, Switzerland).

**
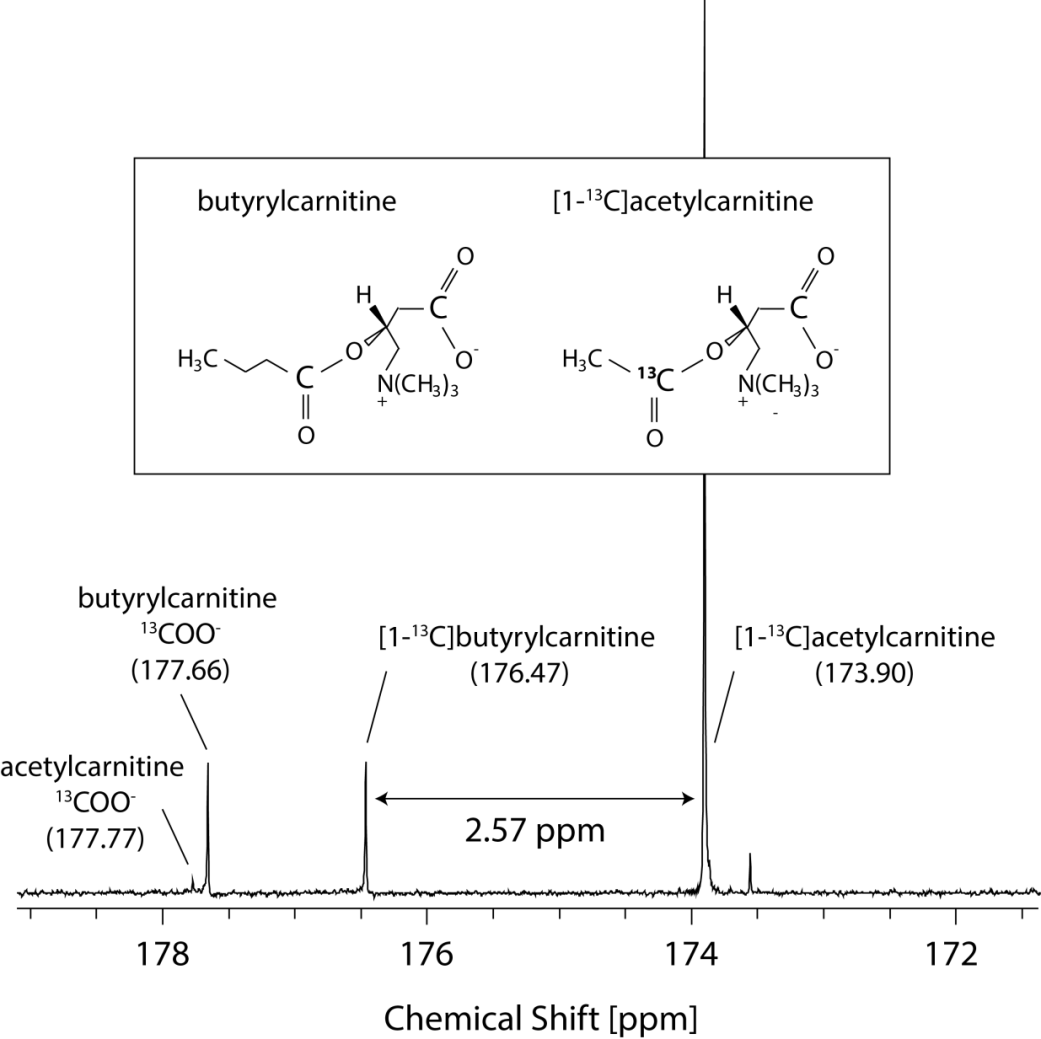
**

**Supplemental Figure S3.** Total ^13^C metabolites relative to the total detected signal derived from pyruvate (left panel) and butyrate (right panel) metabolism. The total ^13^C signal was not significantly different across the groups as analyzed using ANOVA. A trend towards increased signal from pyruvate derived metabolites when butyrate is co-injected did not reach the level of significance.

**
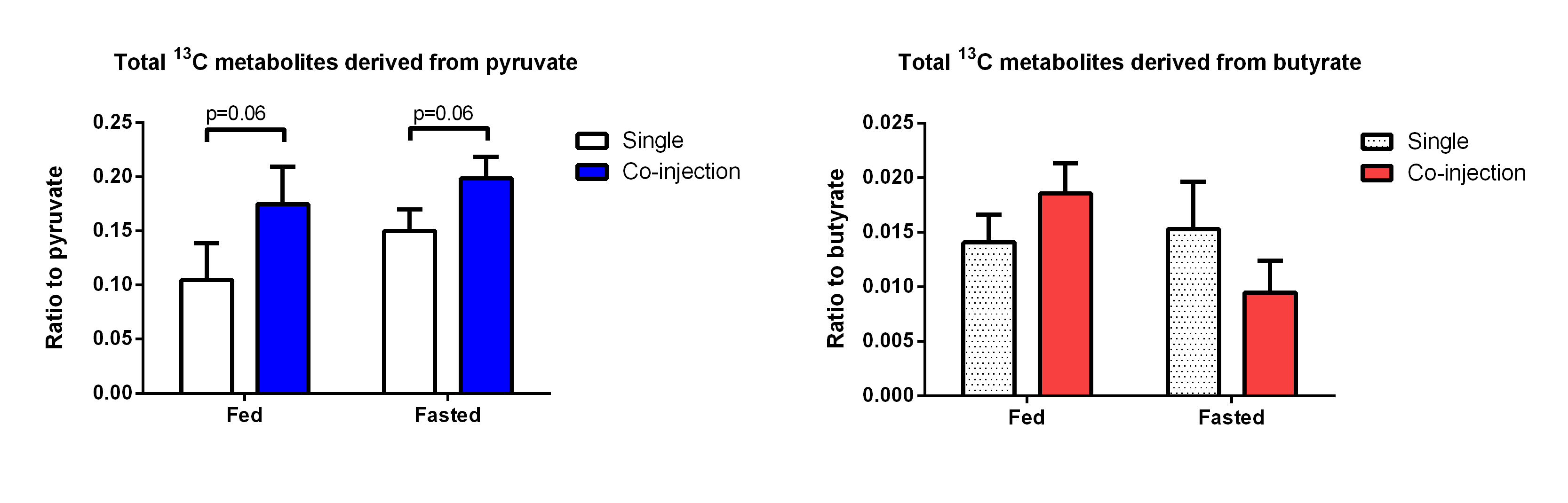
**

**SUPPLEMENTAL DISCUSSION**

***Pyruvate metabolism***

Bicarbonate to pyruvate ratios (~3%) were consistent with previous *in vivo* studies in fed animals . The reduction of the bicarbonate intensity after overnight fasting was less pronounced in our experiments (declined ~50%) compared to [^3^](#_ENREF_3) where a reduction of ~70% was observed. This is likely due to the combination of differences in the fasting protocol or strain of the animals (previous work was done in Wistar rats). Significant changes in lactate to alanine ratios were observed in the fed versus fasted state, as reported for the liver and skeletal muscle as well . The linkage of the [pyr]/[lactate] ratio to the [NAD^+^]/[NADH] ratio in the cytosol has long been understood [^7^](#_ENREF_7), but there can be a disconnect between enrichment and pool sizes in the myocardium [^8^](#_ENREF_8). While it is tempting to interpret these results as a change in redox state, further experiments measuring fractional enrichments in the alanine and lactate pools will be necessary to confirm this interpretation. In summary, the [1-^13^C]pyruvate injections produced responses that would be expected given previous work in the literature.

**REFERENCES**

1. Schroeder MA, Swietach P, Atherton HJ, Gallagher FA, Lee P, Radda GK, Clarke K and Tyler DJ. Measuring intracellular pH in the heart using hyperpolarized carbon dioxide and bicarbonate: a 13C and 31P magnetic resonance spectroscopy study. *Cardiovascular research*. 2010;86:82-91.

2. Gallagher FA, Kettunen MI, Day SE, Hu DE, Ardenkjaer-Larsen JH, Zandt R, Jensen PR, Karlsson M, Golman K, Lerche MH and Brindle KM. Magnetic resonance imaging of pH in vivo using hyperpolarized 13C-labelled bicarbonate. *Nature*. 2008;453:940-3.

3. Schroeder MA, Cochlin LE, Heather LC, Clarke K, Radda GK and Tyler DJ. In vivo assessment of pyruvate dehydrogenase flux in the heart using hyperpolarized carbon-13 magnetic resonance. *Proc Natl Acad Sci U S A*. 2008;105:12051-6.

4. Janich MA, Menzel MI, Wiesinger F, Weidl E, Khegai O, Ardenkjaer-Larsen JH, Glaser SJ, Haase A, Schulte RF and Schwaiger M. Effects of pyruvate dose on in vivo metabolism and quantification of hyperpolarized (13) C spectra. *NMR in biomedicine*. 2012;25:142-51.

5. Merritt ME, Harrison C, Sherry AD, Malloy CR and Burgess SC. Flux through hepatic pyruvate carboxylase and phosphoenolpyruvate carboxykinase detected by hyperpolarized 13C magnetic resonance. *Proceedings of the National Academy of Sciences of the United States of America*. 2011;108:19084-9.

6. Bastiaansen JAM, Yoshihara HAI, Takado Y, Gruetter R and Comment A. Hyperpolarized 13C lactate as a substrate for in vivo metabolic studies in skeletal muscle. *Metabolomics*. 2014;10:986-994.

7. Williamson DH, Lund P and Krebs HA. The redox state of free nicotinamide-adenine dinucleotide in the cytoplasm and mitochondria of rat liver. *The Biochemical journal*. 1967;103:514-27.

8. Purmal C, Kucejova B, Sherry AD, Burgess SC, Malloy CR and Merritt ME. Propionate stimulates pyruvate oxidation in the presence of acetate. *Am J Physiol Heart Circ Physiol*. 2014;307:H1134-41.
